# Systems Toxicology Approach for Assessing Developmental Neurotoxicity in Larval Zebrafish

**DOI:** 10.3389/fgene.2021.652632

**Published:** 2021-06-15

**Authors:** Roman A. Li, Marja Talikka, Sylvain Gubian, Colette vom Berg, Florian Martin, Manuel C. Peitsch, Julia Hoeng, Anze Zupanic

**Affiliations:** ^1^Eawag, Dübendorf, Switzerland; ^2^PMI R&D, Philip Morris Products S.A., Neuchâtel, Switzerland; ^3^National Institute of Biology, Ljubljana, Slovenia

**Keywords:** systems toxicology, zebrafish, developmental neurotoxicity, klf8, rb1, tp53

## Abstract

Adverse outcomes that result from chemical toxicity are rarely caused by dysregulation of individual proteins; rather, they are often caused by system-level perturbations in networks of molecular events. To fully understand the mechanisms of toxicity, it is necessary to recognize the interactions of molecules, pathways, and biological processes within these networks. The developing brain is a prime example of an extremely complex network, which makes developmental neurotoxicity one of the most challenging areas in toxicology. We have developed a systems toxicology method that uses a computable biological network to represent molecular interactions in the developing brain of zebrafish larvae. The network is curated from scientific literature and describes interactions between biological processes, signaling pathways, and adverse outcomes associated with neurotoxicity. This allows us to identify important signaling hubs, pathway interactions, and emergent adverse outcomes, providing a more complete understanding of neurotoxicity. Here, we describe the construction of a zebrafish developmental neurotoxicity network and its validation by integration with publicly available neurotoxicity-related transcriptomic datasets. Our network analysis identified consistent regulation of tumor suppressors p53 and retinoblastoma 1 (Rb1) as well as the oncogene Krüppel-like factor (Klf8) in response to chemically induced developmental neurotoxicity. The developed network can be used to interpret transcriptomic data in a neurotoxicological context.

## Introduction

The developing brain is particularly vulnerable to drugs and environmental chemicals (Rice and Barone, 2000). Developmental exposure to polycyclic aromatic hydrocarbons, pesticides, heavy metals, flame retardants, pharmaceuticals, and other chemicals in private and commercial use has been linked to neurodevelopmental disorders in humans and in animal models (Braun et al., 2006; Barlow et al., 2007; Dufour-Rainfray et al., 2011; Bellinger, 2013). Guidelines for testing the developmental neurotoxicity (DNT) of chemicals, provided by the Organisation for Economic Co−operation and Development (OECD) (OECD, 2007) and the United States Environmental Protection Agency (US EPA) (EPA, 1996), recommend *in vivo* rodent experiments. Although the recommended tests are reliable and reproducible (Makris et al., 2009), testing each chemical requires up to a year of research and up to a million dollars in funding (Crofton et al., 2012). Consequently, the number of chemicals tested for DNT is low (Makris et al., 2009) and represents only a small fraction of the thousands of chemicals in commercial use (Crofton et al., 2012). The need for fast, cheap, efficient screening methods that would help fill in these data gaps is apparent (Fritsche et al., 2018b). *In vitro* cell culture methods are fast, relatively inexpensive, and very useful in studying the mechanisms of DNT (Bal-Price et al., 2010). However, their use obligates one to simplify the central nervous system (CNS) to a single or few cell types in culture. In losing the complexity of interactions between cell types, anatomical regions, and developmental stages, it becomes challenging to translate *in vitro* data into *in vivo* toxicity (Lein et al., 2005). The zebrafish (*Danio rerio*) larva is an alternative model that can be used to complement the *in vitro* and rodent approaches in studying DNT.

The zebrafish is a model organism that has long been used by biologists to study the development and function of the brain (Engert and Wilson, 2012; Haesemeyer and Schier, 2015). This model organism possesses numerous advantages for DNT assessment. Zebrafish and mammals show conserved expression of neurodevelopmental genes (Wullimann, 2009), structural and functional homology of the brain (Tropepe and Sive, 2003), conserved neuronal subtypes and neurotransmitters (Nishimura et al., 2015), and similar behavioral responses to drugs (Rihel and Schier, 2012). Combined with the high practicality of its use (Kalueff et al., 2014) and its rich behavioral repertoire that can be used as a readout for altered brain function (Kalueff et al., 2013), the zebrafish is becoming an increasingly popular model for toxicological assessment of new drug candidates and environmental chemicals (Scholz et al., 2008; Fitzgerald et al., 2020).

Another advantage of the zebrafish is the quality of its genome sequence and annotation (Howe et al., 2013). Combined with high-throughput unbiased molecular methods (such as transcriptomics analyses) and computational analysis (together termed toxicogenomics or systems toxicology), the zebrafish is a great tool for discovery of molecular mechanisms underlying drug- and chemical-induced toxicity (Alexander-Dann et al., 2018). Systems toxicology approaches, such as reverse causal reasoning (Catlett et al., 2013)—which is based on integrating curated biological networks, publicly available omics datasets, and omics data obtained from toxicological experiments—have been shown to be particularly effective for evaluating and predicting chemical toxicity (Hoeng et al., 2012). In this approach, nodes within curated biological networks cover multiple levels of biological organization (e.g., RNA, protein, biological process, and pathology), and edges represent various relationships (e.g., increases, decreases, physical interaction, and association). This flexibility allows for a realistic representation of biology. Additionally, the edges can be directed, which gives the networks a cause-and-effect topology. This feature aids in following the propagation of the signal through the network, from molecular initiating events to adverse outcomes. The networks can be tailored to a particular set of related adverse outcomes within specific organs or organ systems and, therefore, allow focused toxicological interpretation. At the same time, if multiple curated networks are available, a single transcriptomics experiment allows one to evaluate the toxicity to different tissues, organs, or organ systems at once (Hoeng et al., 2014). This approach can provide information on the probability that a specific organ toxicity is induced and on the main molecular events responsible for it. This feature is particularly useful with small organisms, such as larval zebrafish, when single organs often provide insufficient material for transcriptomic analysis, and the whole animal is sequenced.

Although the number of curated biological networks describing toxicity in humans, rats, and mice is steadily growing (Boue et al., 2015; Yepiskoposyan et al., 2019), the current lack of such networks for zebrafish precludes large-scale systems toxicology assessment of zebrafish omics data. To begin addressing this paucity, we recently constructed and validated the first zebrafish network for describing cardiac toxicity (Li et al., 2020). We have shown that network scoring can predict toxicity days before the onset of visible cardiac phenotypes and identified candidate molecular events that mediate cardiac toxicity. Here, we describe the construction, validation, and applicability of the second zebrafish network, which describes neurotoxicity in developing larvae.

## Materials and Methods

### Curation and Compilation of the Zebrafish Neurotoxicity Network

The neurotoxicity network was constructed by converting causal molecular relationships reported in literature into the Biological Expression Language (BEL; version 1.0)^1, 2^. BEL is used to represent biological knowledge in a computable form as BEL statements. Each BEL statement consists of a source node, a target node, and a relationship between them (called an edge). Nodes in BEL can represent multiple levels of biological organization, such as RNA, protein, protein activity, biological process, or pathology. BEL uses controlled vocabularies (namespaces) for node names; for example, human genes and proteins are labeled in accordance with the HUGO Gene Nomenclature Committee (HGNC) vocabulary, and biological processes follow the Gene Ontology Biological Process (GOBP) vocabulary. This ensures uniformity of language and enables computation. Edges can represent various regulation by the source node, e.g., inhibition, activation, and translation. Additionally, each BEL statement is annotated with metadata (e.g., publication, species, tissue, and experimental methods) to facilitate future verification. We used the open source framework OpenBEL 3.0.0^3^ to compile curated BEL statements into a connected network. Supplementary Table 1 lists all BEL statements and may be used to create an interactive version of the network. We used Cytoscape (Shannon et al., 2003) to visualize and analyze the network. Alternatively, an interactive version of the network is available at http://causalbionet.com/.

### Network Scoring

**FIGURE 1:**
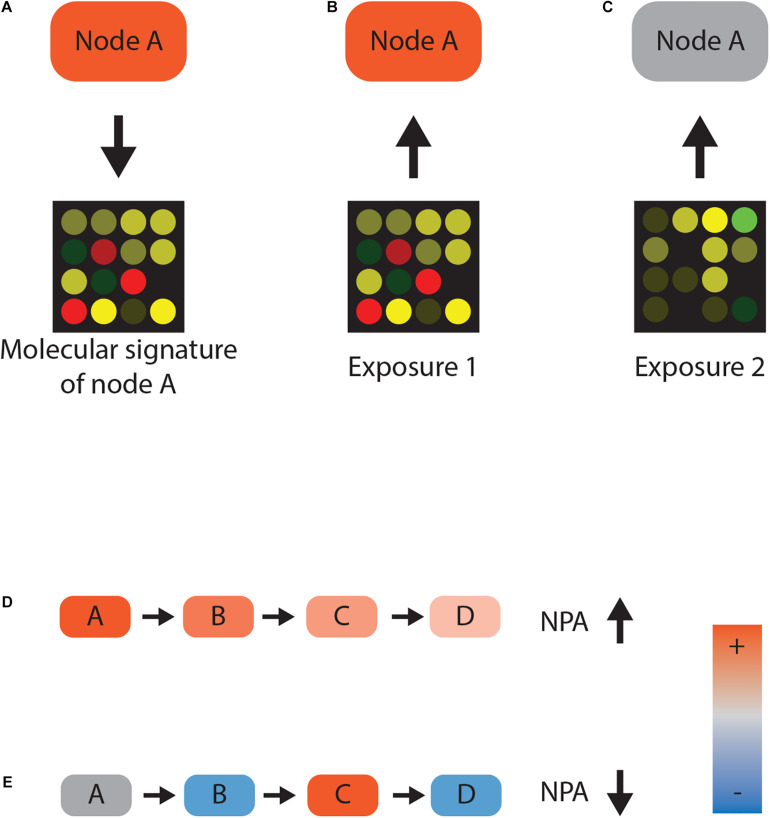
Network scoring. **(A–C)** Reverse causal reasoning paradigm. Gene expression data following activation of protein A can be used as a molecular signature for the activity of that protein A. Any given transcriptomic dataset—for example, from chemically exposed samples—can now be used to infer the activity of protein A. Closely matching gene expression values indicate that protein A was active **(B)**, whereas disparate gene expression is not consistent with the activity **(C)**. **(D,E)** Network perturbation amplitude (NPA) calculation. If nodes A, B, and C positively regulate node D and their activities are inferred to be high, there is a high consistency between gene expression and network topology. High consistency leads to a high NPA value **(D)**. Conversely, if node activity and the topology of the network contradict each other, the NPA value decreases **(E)**.

An important part of our approach is integration of the network with high-throughput omics measurements to evaluate the (neuro) toxicity of chemicals, as described in detail recently by Martin et al. (2019). The central idea is that experimentally obtained omics (usually transcriptomic) data can be used to infer activity of upstream molecular events (Figures 1A–C). Nodes with a known differential gene expression signature are called inferable nodes (iNodes). Mapping gene expression molecular signatures to iNodes creates an additional layer of information. Therefore, literature-based statements represent the functional layer, and the transcriptomic signatures of the iNodes in the functional layer represent the transcript layer. The network scoring algorithm works by first calculating the activity value (similarity between the query gene expression and gene expression from the transcript layer) for each iNode. Then, for each node in the functional layer (both iNodes and regular nodes), the algorithm calculates a node coefficient, which takes into account the inferred activity values of the iNodes and the topology of the network (Figures 1D,E). Inferred activities that are well accommodated by the functional layer result in high node coefficients and vice versa. The node coefficients are then used to calculate the network perturbation amplitude (NPA). The NPA can be used as a measure of disruption specific to the network scored. For biological networks that describe organ toxicity, the NPA can be used to quantify toxicity specific to that organ. The NPA is reported with confidence intervals and two companion statistics: “o” and “k.” Confidence intervals are calculated on the basis of the assumption that gene expression values in the transcript layer follow normal distribution and provide information on the variance of the NPA. The “o” statistic is calculated by permuting the edges in the transcript layer and recalculating the NPA value. The “k” statistic is calculated by permuting the edges in the functional layer and recalculating the NPA value. In both cases this procedure is performed 500 times and statistical significance is reached if the original NPA is in the top 5th percentile of the permuted NPA. Therefore, the two statistics test the null hypothesis that a random arrangement of the transcription layer or the functional layer can produce a result similar to the original NPA. If 95% of the time this is false, we conclude that the dataset being scored specifically perturbs the two layer network, and this result is not due to random chance (Martin et al., 2014).

### Leading Node Analysis

We performed leading node (LN) analysis to identify regions of the network responsible for the observed impact and to gain insights into the underlying mechanisms. LNs are nodes that are responsible for 80% of the total NPA score and are, therefore, the major drivers of network perturbation. Nodes contribute more to the total NPA score if their inferred activity is highly impacted and consistent with the neighboring nodes in the functional layer. This analysis has been shown to be a useful way for interpreting the biological basis of network perturbations (Hoeng et al., 2012; Martin et al., 2014). In addition, LN analysis indicates the directionality of the impact on each node. The entire network scoring algorithm, including LN analysis, is described in detail by Martin et al. (2019) and is available on the GitHub project pages https://github.com/pmpsa-hpc/NPA and https://github.com/pmpsa-hpc/NPAModels as an R package. The package includes information on the transcript layer used to score the network in this paper. This will allow interested researchers to replicate our results or try our approach on their own transcriptomic datasets. In our figures, LNs are color-coded to help visualize signal propagation through the network and to identify the most impacted areas. Activated nodes are red, while inactivated nodes are blue, and unaffected nodes are gray. The heatmap was scaled to minimum and maximum activation for each dataset independently but was not altered between treatments in individual datasets.

## Results

### Zebrafish Neurotoxicity Network

To construct the zebrafish neurotoxicity network, we curated peer-reviewed articles that describe adverse neural outcomes in zebrafish larvae. We searched PubMed^4^ for articles containing the keywords “zebrafish larvae” and one of the following neurotoxic phenotypes commonly reported in zebrafish: “microcephaly” (reduced brain size), “megalencephaly” (enlarged brain size), “microphthalmos” (reduced eye size), “hydrocephalus” (accumulation of fluid around the brain), “seizures,” “neurogenic inflammation,” “sleep,” “phototaxis,” or “locomotion.” We collected data-supported mechanistic findings from these papers and converted them into computable statements. For example, the natural language sentence: “*As shown in Figure 13, exposure to 2% ethanol or treatment with agrin or Shh MO results in a significant decrease in brain volume*.” (Zhang et al., 2013) was expressed as three BEL statements:

**a**(CHEBI:ethanol) → **path**(MESHD:Microcephaly)

**act**(**p**(ZFIN:agrn)) -| **path**(MESHD:Microcephaly)

**act**(**p**(ZFIN:shha)) -| **path**(MESHD:Microcephaly)

We then searched literature for molecular interactions upstream and downstream of the initially identified nodes in the zebrafish brain and added these statements to the network. For example, looking for upstream regulators of *shha* in the zebrafish brain we found: “*gbx2 induction*… *moderately upregulated shha in the ventral brain, including the hypothalamus (Figure 3H)*” (Wang et al., 2018). This evidence was added as:

**act**(**p**(ZFIN:gbx2)) → **r**(ZFIN:shha)

**FIGURE 2:**
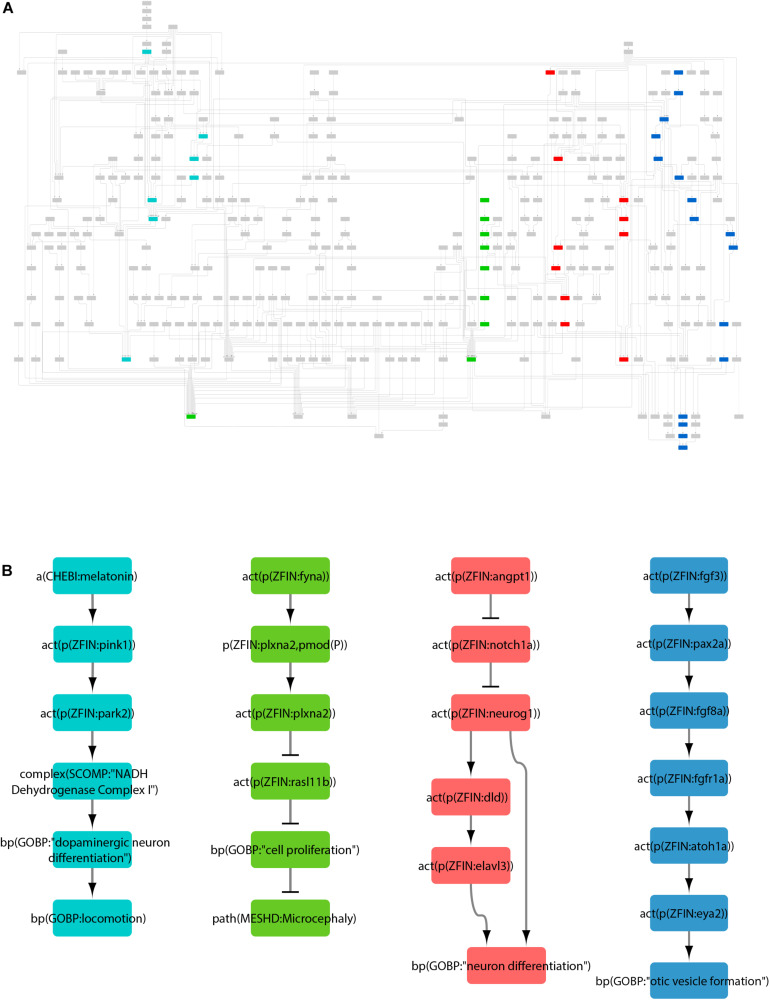
The zebrafish neurotoxicity network. **(A)** The entire network visualized in Cytoscape. **(B)** Selected pathways extracted from the network and color-coded to nodes in panel A. To simplify visualization, RNA, protein, and protein activity nodes were collapsed into a single corresponding node.

With this approach, we curated a total of 87 articles to create the zebrafish neurotoxicity network (Supplementary Table 2). The network contains 344 nodes connected by 479 edges (Figure 2A). Selected pathways were extracted from the network to highlight some of the biological processes and their regulation within the network (Figure 2B). The nodes represent 122 proteins, 118 protein activities, 46 mRNAs, 23 chemicals, 22 biological processes, 6 pathologies, 5 complexes, and 2 microRNAs (Supplementary Table 3). The edges are annotated with metadata that detail the publication and evidence used to create that edge. When available, annotation for species, type of experiment conducted, cell type, confidence, anatomy, and disease are also included (Supplementary Table 1). An interactive version of the network model can be accessed on http://causalbionet.com. Nodes with many interactions are said to have a high node degree and are referred to as hubs. The top ten hubs with the highest node degrees are listed in Table 1. Nodes with high betweenness centrality are referred to as bottlenecks (Yu et al., 2007) and are listed in Table 2.

**TABLE 1:** Nodes with the highest number of total edges.

Node name	Edge count
bp(GOBP:”cell proliferation”)	19
bp(GOBP:”cell death”)	16
bp(GOBP:”dopaminergic neuron differentiation”)	11
m(ZFIN:”mir9-2”)	5
act(p(ZFIN:lef1))	5
p(ZFIN:lef1,pmod(P))	5
bp(GOBP:”apical junction assembly”)	4
act(p(ZFIN:tp53))	4
act(p(ZFIN:plxna2))	4
act(p(ZFIN:notch1a))	4

**TABLE 2:** Nodes with the highest betweenness centrality.

Node name	Betweenness centrality
act(p(ZFIN:pax2a))	0.00927118
act(p(ZFIN:neurog1))	0.00612848
bp(GOBP:”cell proliferation”)	0.00368594
p(ZFIN:dld)	0.00332378
p(ZFIN:her5)	0.00308637

### Network Scoring With Public Datasets

**FIGURE 3:**
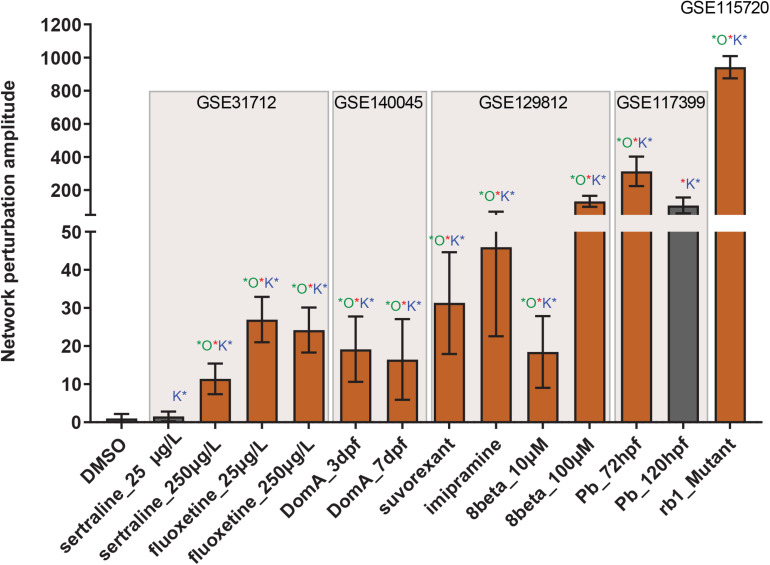
Neurotoxicity network perturbation amplitude (NPA). NPA values are shown as fold change over DMSO control with proportional adjustment of confidence intervals. Beige rectangles highlight separate transcriptomic datasets. Red stars indicate significant NPA values. Green stars indicate significant o statistic values — a measure of how specific the given perturbation is to the current network. Blue stars indicate significant o statistic values — a measure of the quality of the functional layer. Orange bars indicate significant perturbation, while gray bars represent values where at least one statistic was not significant.

To score the network with transcriptomic data, we searched the Gene Expression Omnibus repository^5^ for datasets with differentially expressed genes (DEG) from experiments that involved genetic or chemical manipulation of the zebrafish central nervous system. We additionally constrained the search to larval stages of the zebrafish, as the network was curated using data obtained from 3 to 8 dpf zebrafish, and to datasets that had three replicates per condition available, in order to perform statistics. We selected datasets GSE55618, GSE115720, GSE31712, GSE140045, GSE129812, and GSE117399. Dataset GSE55618 (Driessen et al., 2015) was used as a negative control, as the authors had submitted data both for distilled water and 0.2% dimethyl sulfoxide (DMSO) treatment. At this low concentration, DMSO is not expected to be neurotoxic (Christou et al., 2020). In accordance, scoring with this dataset did not perturb the network, and all the following values were normalized to those of DMSO treatment (Figure 3).

We first used dataset GSE115720 (Schultz et al., 2018). In this study, the authors sequenced the mRNA of the zebrafish larval heads, either wildtype or homozygous mutant for *retinoblastoma 1* (*rb1*). Rb1 is a tumor suppressor protein that exerts its function by regulating cell proliferation, and genetically *rb1*-mosaic adult zebrafish develop brain tumors (Solin et al., 2015). Although DNT is usually associated with chemical exposure, we selected *rb1* mutants as a positive control because the larvae displayed a clear neurological phenotype and had a defined genetic background, thus providing a link between the mutation and the phenotype. The transcriptome of the *rb1*^–/–^ larvae elicited the biggest network perturbation of all treatments (Figure 3).

In GSE31712, the authors explored the effects of two selective serotonin reuptake inhibitors (SSRI), sertraline and fluoxetine, on whole larvae at 25 and 250 μg/L (Park et al., 2012). SSRIs are prescription drugs used to treat clinical depression and have been found to have neuroactive effects in fish (Kreke and Dietrich, 2008). Both drugs elicited statistically significant network perturbations at the higher concentration, while only fluoxetine resulted in a significant perturbation at the lower dose (Figure 3).

Dataset GSE117399 considered the effects of lead (Pb) on the larval nervous system (Peterson et al., 2011), as Pb exposure is associated with DNT, neuronal cell death, excitotoxicity, and behavioral defects in children (Sanders et al., 2009). The authors analyzed the transcriptomes of whole larvae treated with 100 ppb Pb after 72 and 120 h of exposure. The network was highly perturbed at both time points, but the “o” statistic (which measures the adequacy of assignment of the transcript layer genes) did not reach statistical significance at 120 post-fertilization (hpf) (Figure 3).

We also scored the network with two datasets which, at the time of writing, did not have associated publications. One of the studies (GSE140045) evaluated the effects of domoic acid (Domo)—a potent neurotoxin produced by harmful algal blooms—on whole larvae at two different time points. In the second dataset (GSE129812), larvae were exposed to insomnia medication suvorexant, antidepressant imipramine, and a plant-derived orexin antagonist 8β-(4′-hydroxytigloyloxy)costunolide (8beta). The latter chemical was used at two concentrations. The brains of these larvae were used for microarray. All of the above chemicals have neuroactive properties in mammals. In accordance, the transcriptome of larvae treated with these chemicals showed significant perturbation of the neurotoxicity network (Figure 3).

### LN Analysis

**FIGURE 4:**
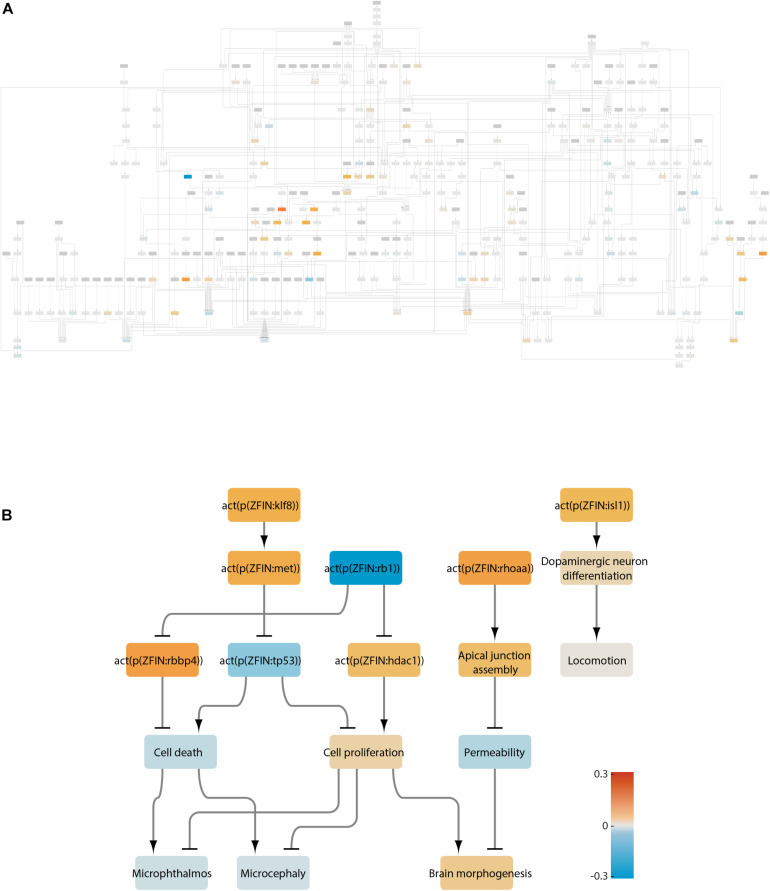
Neurotoxicity network scored with the transcriptome of rb1 mutant larvae. **(A)** Visualization of the affected nodes in the network. Increased and reduced activity for each node is color-coded according to the heatmap. **(B)** Leading nodes extracted from A into a subnetwork.

To gain mechanistic insights, we performed LN analysis for the datasets that produced a significant NPA. In the transcriptome of the *rb1*^–/–^ larval heads, thirteen LNs (i.e., nodes that together contribute up to 80% of the NPA) were identified (Figure 4A), color-coded, and extracted into a smaller subnetwork (Figure 4B). For ease of interpretation, relevant downstream adverse outcomes and biological processes were added to the subnetwork independently of whether they were identified as LNs. Reassuringly, the top inferred inactivated LN in the *rb1*^–/–^ dataset was Rb1. This was followed by downstream activation of two chromatin remodeling proteins, histone-binding protein Rbbp4 and histone deacetylase Hdac1, and led to increased cell proliferation and decreased cell death. Proliferation and cell death were also impacted by inactivation of the tumor suppressor p53 downstream of Klf8 (Krüppel-like factor 8) and Met (MET proto-oncogene, receptor tyrosine kinase). These computational results are consistent with the findings of the original study, where the authors reported increased cell proliferation in the developing brain and retina of the mutant larvae and altered p53 signaling. In addition to the original findings, the network approach inferred positive regulation of the apical junction assembly by Rhoaa (ras homolog gene family, member Aa). Together, the node activities converge on microphthalmos, microcephaly, and brain morphogenesis, but none is significantly affected. This finding fits well with the phenotype of *rb1*-/- larvae, which do not show gross morphological differences when compared with their wildtype siblings (Schultz et al., 2018). Network scoring also suggested a possible effect on dopaminergic neuron differentiation due to increased activity of Isl1 (ISL LIM homeobox 1); however, locomotion downstream of these nodes was not affected.

**FIGURE 5:**
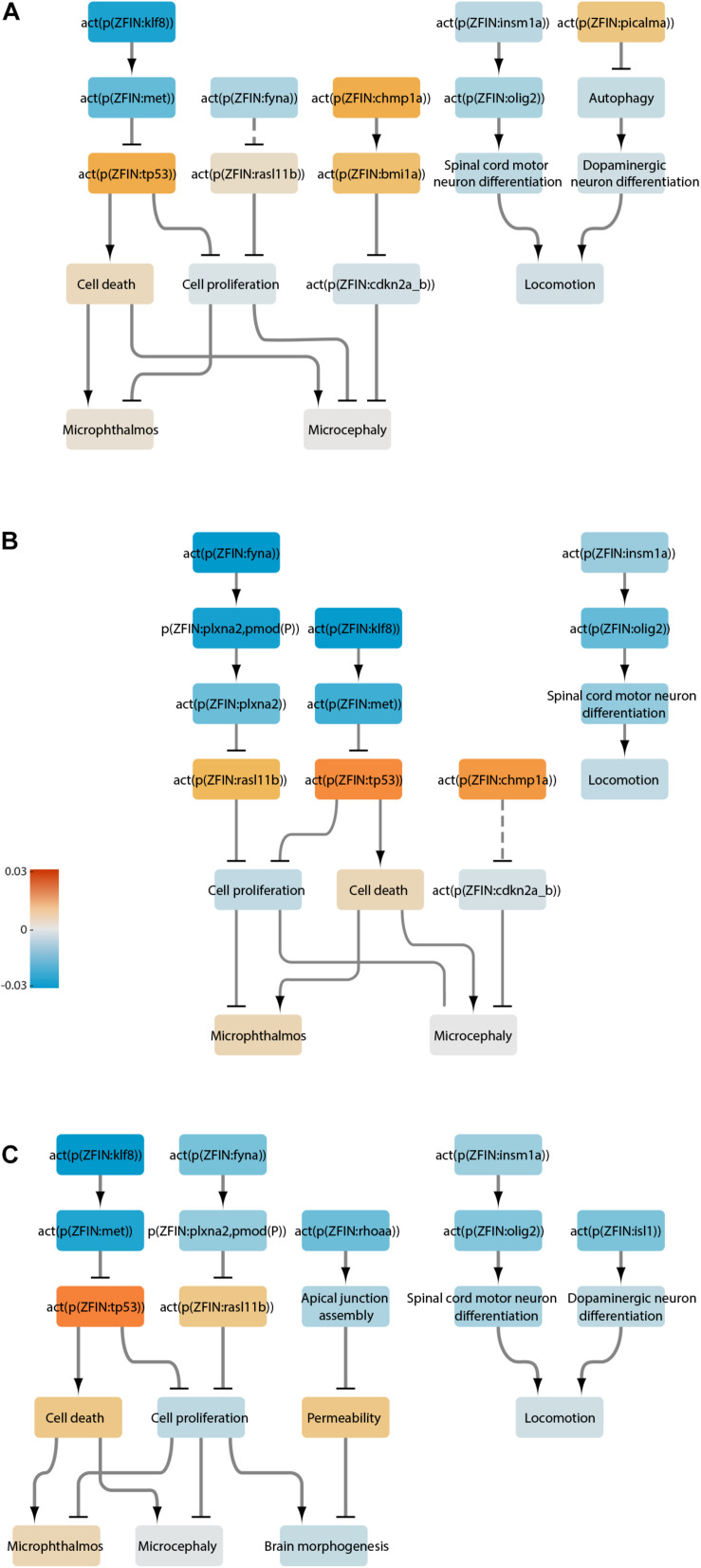
Neurotoxicity network scored with the transcriptomes of larvae treated with selective serotonin reuptake inhibitors. **(A)** Sertraline treatment, 250 μg/L. **(B)** Fluoxetine, 25 μg/L. **(C)** Fluoxetine, 250 μg/L. Increased and reduced activity for each node is color-coded according to the heatmap. Dotted lines represent indirect connections in the main network.

We next analyzed the SSRIs sertraline and fluoxetine. Because sertraline at 25 μg/L did not elicit a significant network perturbation, we excluded this treatment from further analysis. Sertraline at 250 μg/L caused a strong inferred downregulation of Klf8, which led to activation of p53 signaling in the network (Figure 5A). The subnetwork also indicated a modest increase in the activity of Chmp1a (charged multivesicular body protein 1a) and Bmi1a (bmi1 polycomb ring finger oncogene 1a), followed by reduced activity of the negative regulator of proliferation, Cdkn2a/b (cyclin dependent kinase inhibitor 2A/B). Additionally, we inferred reduced activity of Fyna (FYN proto-oncogene, Src family tyrosine kinase a), a protein that plays a role in synaptic function (Nygaard et al., 2014), and increased activity of the downstream small GTPase Rasl11b (RAS-like, family 11, member B). Although the above nodes play roles in cell proliferation and apoptosis, the topology of the network, together with node activity, did not suggest a significant effect in either process. However, network scoring indicated a reduction in motor neuron differentiation in the spinal cord due to a decrease in the activity of Olig2 (oligodendrocyte lineage transcription factor) and a decline in dopaminergic neuron differentiation due to increased activity of the synaptic vesicle-associated Picalma (phosphatidylinositol binding clathrin assembly protein A) and reduced autophagy. Together, these processes led to a marginal reduction in node “locomotion”; however, this effect was not significant.

At both 25 and 250 μg/L, fluoxetine treatment elicited very similar effects to sertraline (Figures 5B,C). These included strongly reduced Klf8 and Met activity and activation of p53; reduced Fyna activity, reduced Plxna2 (plexin A2; axon guidance protein) phosphorylation and activity, and increased Rasl11b activity; and reduced motor neuron differentiation due to reduced Olig2 and Insm1a (insulinoma-associated 1a) activity. However, the node “locomotion” downstream of these nodes was not affected at either concentration. Chmp1a was inferred to be activated at the lower concentration of fluoxetine, and Isl1 (ISL LIM homeobox 1) was inferred to be inactivated at the higher concentration. Rhoaa and the downstream apical junction assembly were affected at 250 μg/L, suggesting that the integrity of the neurepithelium may be affected at this or higher concentrations. Both concentrations resulted in a small but significant reduction in the node “cell proliferation,” and 250 μg/L fluoxetine caused a modest increase in the node “cell death,” suggesting that the higher dose is more neurotoxic. However, none of the pathologies in the network were affected at either concentration. This result is consistent with the absence of changes in mortality, appearance, or behavior in the treated larvae (Park et al., 2012).

**FIGURE 6:**
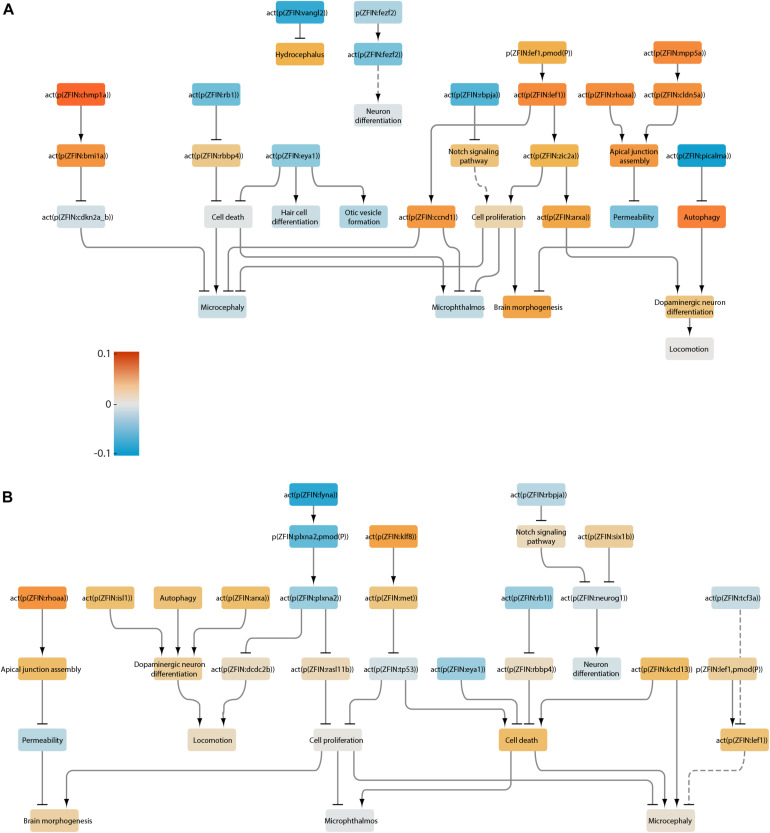
Neurotoxicity network scored with the transcriptomes of larvae treated with lead. **(A)** 72-hour exposure. **(B)** 120-hour exposure. Increased and reduced activity for each node is color-coded according to the heatmap. Dotted lines represent indirect connections in the main network.

The subnetworks in Pb-treated larvae revealed some similarities as well as marked differences in the 72- and 120-hour treatments (Figure 6). In both cases, the following inferences were made: reduced activity of Rb1, Eya1 (EYA transcriptional coactivator and phosphatase 1), and Rbpja (recombination signal binding protein for immunoglobulin kappa J region a) upstream of Notch signaling; increased activity of Lef1 (lymphoid enhancer-binding factor 1), Rhoaa upstream of apical junction assembly; and increased autophagy, with the 72-hour group showing a greater predicted increase out of the two treatments. For the 72-hour Pb exposure (Figure 6A), the subnetwork predicted the following: activation of Mpp5a (membrane protein, palmitoylated 5a) and Cldn5a (claudin 5a), suggesting that this treatment might affect epithelium integrity; increased activity of Chmp1a and Bmi1a; increased activity of Ccnd1 (cyclin-D1) and cell proliferation; decreased activity of Picalma; decreased activity of Fezf2 (FEZ family zinc finger 2) upstream of neuron differentiation; and decreased activity of Vangl2 (VANGL planar cell polarity protein 2) upstream of hydrocephaly. For the 120-hour Pb exposure (Figure 6B), the following changes were predicted: reduced activity or Neurog1 (neurogenin 1) and inhibition of downstream neuronal differentiation; activation of Klf8; reduced activity of Fyna and Plxna2; activation of Kctd13 (potassium channel tetramerization domain containing 13); and an increase in the node “cell death.”

For exposure to 0.14 ng of Domo at two developmental windows, we inferred strongly activated Klf8 and inactivated p53 (Supplementary Figure 1A); activated dopamine receptors D2a (Drd2a) and D2b (Drd2b); and inactivated Kctd13 and Ebf2 (a proneuronal transcription factor). Exposure at 3 days post-fertilization (dpf) resulted in inactivation of Elavl3 (ELAV like neuron-specific RNA binding protein 3; a proneuronal protein) and Neurog1 and increase in Ccnd1 activity. These results suggest that Domo treatment at 3 dpf has an inhibitory effect on neurogenesis and favors a proliferative state. Lastly, the LNs also suggest an increase in the activity of Lgi1a and 1b (leucine-rich glioma-inactivated protein 1a and 1b), which is associated with epileptic seizures (Cowell, 2014). Domo exposure at 7 dpf resulted in inhibition of Mpp5a, Chmp1a, and Bmi1a upstream of Cdkn2a/b, as well as inhibition of Chd8 (chromodomain helicase DNA binding protein 8) (Supplementary Figure 1B). Increased autophagy and increased activity of NADH dehydrogenase complex I and Isl1 positively regulated the node “dopaminergic neuron differentiation.” Lastly, the activity of the epilepsy-associated protein Tsc2 (TSC complex subunit 2) was decreased in this group.

In the dataset GSE129812, suvorexant, imipramine, and 8beta reduced the activity of Klf8 and Met and increased the activity of p53. Suvorexant exposure resulted in marginally and not significantly increased cell death via increased activity of p53 and Kctd13, activated Mpp5a and Cldn5a, and decreased activity of proteins involved in neuron differentiation (Olig2, Fezf2, and Neurog1), planar cell polarity (Vangl2), axon guidance (Fyna and Plxna2), and otic vesicle formation (Eya1) (Supplementary Figure 2A). Inactivation of both Tsc2 and Lgi1a suggested an increase in the node “seizures.” Imipramine treatment increased the activity of tumor suppressors (Rb1 and p53), autophagy, and proteins involved in embryonic development [Fgf3 (fibroblast growth factor 3), Fgf8a (fibroblast growth factor 8a), Pax8 (paired box 8), and Pax2a (paired box 2a)] and increased cell death (Supplementary Figure 2B). Suvorexant and imipramine had opposite effects on the activity of the Rb1 node, resulting in a significant increase in the node “cell death” in imipramine-treated larvae.

Exposure to 10 and 100 μM 8beta resulted in largely similar outcomes, with the higher concentration producing a greater effect (Supplementary Figure 3). Reduced activity of Vangl2 suggested increased hydrocephaly at both concentrations, and this increase became significant at 100 μM. Increased activity of proteins crucial for CNS development (Fgf3, Pax2a, Wnt1, and Lef1) suggests that the nervous system developed. However, increased activity of Kctd13 and tumor suppressors p53 and Rb1 indicate an increase in cell death at the highest concentration of 8beta. Interestingly, ethanol activity was inferred to be upregulated, suggesting that 8beta and ethanol result in somewhat similar gene expression signatures in the developing CNS.

**FIGURE 7:**
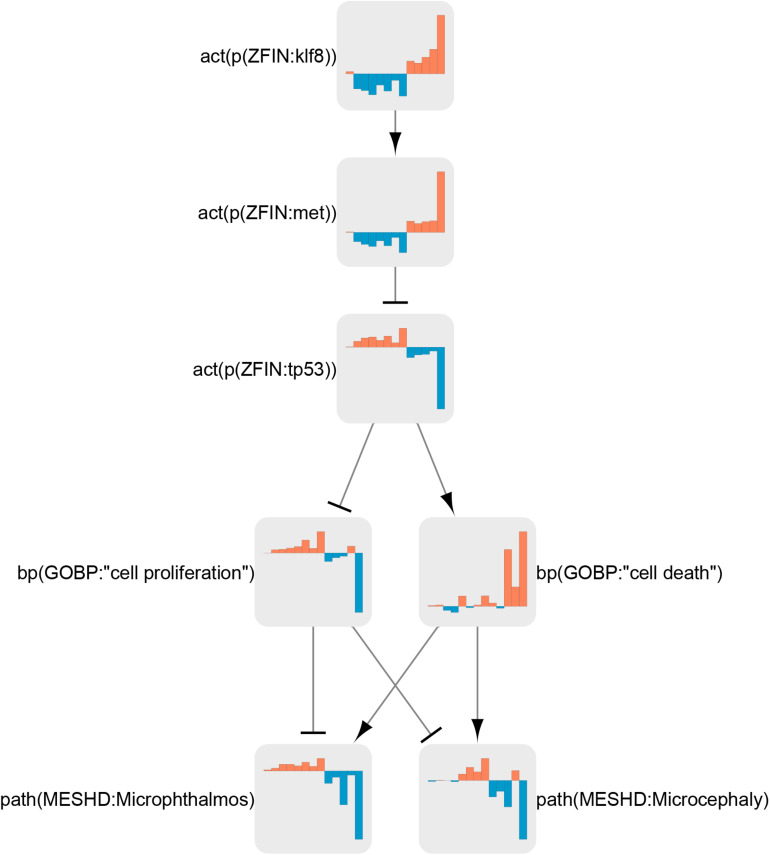
Klf8 regulation in the neurotoxicity network. Node coefficient values (see section “Materials and Methods”) for all treatments are shown as bar graphs. Bars from left to right correspond to neuroactive (ser 25 μg/L, ser 250 μg/L, flu 25 μg/L, flu 250 μg/L, suvo, imip, 8beta 10 μM, and 8beta 100) and neurotoxic (domo 3 dpf, domo 7 dpf, Pb 72 hpf, Pb 120 hpf, and rb1) conditions. Orange bars indicate activation, and blue bars indicate inactivation.

The node “Klf8” was significantly regulated in all exposures apart from the low concentration of sertraline (Figure 7). Klf8 was inactivated in antidepressant and insomnia treatments (sertraline, fluoxetine, suvorexant, imipramine, and 8beta) and strongly activated in neurotoxic conditions (Domo, Pb, and *rb1* mutants). This was followed by changes in the activity of the downstream nodes Met, p53, cell death, cell proliferation, microphthalmos, and microcephaly.

## Discussion

Zebrafish are small vertebrates whose larvae are uniquely suited for high-throughput methods, drug and chemical screening, and systems approaches. These characteristics have made them a popular candidate for transcriptomics analysis. In September 2020, the Gene Expression Omnibus (GEO) repository listed 28,199 transcriptomic datasets generated in zebrafish (Barrett et al., 2013). To help leverage this wealth of data, we developed a zebrafish-specific causal biological network as a tool to complement current methods on gene expression analysis. Instead of analyzing individual proteins or discrete pathways, network biology considers systems-level perturbations in response to stress or disease. This helps connect molecular events to biological processes and eventually phenotypes, which, in turn, may help identify therapeutic targets (Barabasi et al., 2011). The network was curated using data obtained from 3 to 8 dpf larval zebrafish – the point at which a lot of neurodevelopmental processes are complete or are in the process of completion. We demonstrate the utility of the network in analyzing transcriptomic datasets generated at these developmental stages (between 3 hpf and 7 dpf) as well as datasets from several tissues (whole larvae, dissected heads, and brains). These qualities should make the network useful for a wide range of basic and applied research using 3–8 dpf zebrafish organs or whole larvae.

### Network Hubs and Bottlenecks

Network hubs are structurally important for the topology of any network, and identification of these highly connected nodes may have practical implications. Hub proteins tend to be encoded by essential genes (Jeong et al., 2001), are preferentially targeted by pathogens (Schleker and Trilling, 2013), and, at least in the case of some cancers, are enriched for genes associated with disease (Jonsson and Bates, 2006). We identified the biological processes cell proliferation, cell death, dopaminergic neuron differentiation, and apical junction assembly as hubs in the network (Table 1). Although this finding is not unexpected, it underscores the importance of these biological processes in mediating neurotoxicity. In agreement, some of these processes were identified as crucial endpoints for DNT assay development (Fritsche et al., 2018a).

Among the proteins identified as hubs were Plxna2— a semaphorin receptor with a well-established role in axon guidance during CNS development (Luo et al., 1993); Plxna2 deficiency has been linked to psychiatric disorders (Wray et al., 2007). The microRNA miR9, another hub in the network, plays important roles in controlling neural progenitor proliferation and migration as well as in maturation of differentiated neurons (Coolen et al., 2013) and has been associated with psychiatric disorders (Tovo-Rodrigues et al., 2019). Another hub, the transcription factor Lef1, is a component of the Wnt/β-catenin pathway and is, therefore, indispensable during CNS development (Noelanders and Vleminckx, 2017). Aberrant regulation of this pathway has been linked to neurodegeneration and mental disorders (Bem et al., 2019). Another hub, notch1a receptor, is a member of the Notch signaling pathway, which can regulate neural stem cell maintenance, proliferation, and apoptosis and is a critical pathway in brain development and neurological disease (Lasky and Wu, 2005). The Wnt and Notch pathways have been reported to crosstalk during CNS development (Ma et al., 2019), and mir9 and Notch interact to control neural progenitor cell proliferation and differentiation (Roese-Koerner et al., 2017). Overall, the neurotoxicity hubs are highly connected within the network and between each other and follow the notion that network hubs tend to be associated with disease (Wachi et al., 2005). Interestingly, the identified neurotoxicity hubs have been specifically associated with schizophrenia (Mah et al., 2006; Panaccione et al., 2013; Topol et al., 2016; Hoseth et al., 2018), suggesting that the network we have developed may be useful for studying this disease. Schizophrenia is a complex psychiatric disorder thought to arise from abnormalities during development of the brain; however, its exact causes are unknown (Weinberger, 1987). Pharmacological and genetic zebrafish models have been developed to help unravel the etiology of the disease (Gawel et al., 2019). It will be interesting to see whether network scoring with the transcriptomes of these models might help identify not only the affected neurodevelopmental processes but also an effective treatment for reversing these changes.

Nodes with high betweenness centrality have the largest number of shortest paths going through them. This topological quality places bottleneck nodes at key points of information flow within the network, which can translate to biological essentiality (Yu et al., 2007). Hub nodes and bottleneck nodes tend to overlap (Lawless et al., 2014). Indeed, cell proliferation was identified in the top ten lists for both methods. Pax2a plays one of the central roles in the development and patterning of the CNS (Thompson and Ziman, 2011) and has the highest betweenness centrality in the neurotoxicity network. Neurog1 is a key transcriptional regulator of neuronal differentiation (Yuan and Hassan, 2014). Her5 (hairy-related 5) and Dld (deltaD) play a role in the Notch signaling pathway, which is a key player in lateral inhibition during neurogenesis (Beatus and Lendahl, 1998). The high betweenness centrality of these developmentally important nodes suggests that they could serve as markers for DNT.

### Network Perturbation Amplitude

We analyzed the transcriptomes of larvae treated with neuroactive chemicals, and all but two treatments caused significant perturbation in the neurotoxicity network, with the positive control Rb1 producing the largest perturbation (over 900-fold greater over the DMSO control). There was a clear dose–response relationship in sertraline- and 8beta-treated larvae but not in those treated with fluoxetine. The higher concentration of fluoxetine (250 μg/L) resulted in fewer DEGs than the lower concentration in the original study, which led the authors to suggest that fluoxetine elicits a non-linear response (Park et al., 2012). Such a response seems to be not uncommon in toxicological literature, with some estimates suggesting that around 20% of dose–response relationships are U-shaped when concentrations below observable toxicity values are used (Calabrese and Baldwin, 2001). The 250 μg/L fluoxetine treatment fits this concentration range, as Park et al. (2012) reported no changes in mortality, morphology, or behavior in the exposed larvae. In contrast, sertraline elicited a lower NPA at 250 μg/L and no response at 25 μg/L. Fluoxetine and its neuroactive metabolites have a half-life of 1–4 days and 7–15 days respectively, while sertraline’s half-life ranges from 22 to 36 h in humans (Altamura et al., 1994; DeVane et al., 2002). As Park et al. (2012), exposed the larvae to both SSRIs for 96 h, it is plausible that sertraline has a shorter half-life in zebrafish, and this translates to lower NPA values. The network was similarly perturbed by Domo at 3 and 7 dpf, suggesting that the larvae are equally sensitive to this neurotoxin at both time points.

Lead exposure produced a significant NPA at 72 and 120 h, but significance for accompanying statistics was not reached at 120 h. In the original study, GO analysis indicated that the 72-hour Pb exposure was strongly associated with neurological disease, whereas the 120-hour exposure affected general development. This suggests that our network can be perturbed by general toxicity; however, the o and k statistics help distinguish this from neurotoxicity. This interpretation, although attractive, should be viewed with caution until more transcriptomes of zebrafish larvae treated with neuroactive as well as generally toxic chemicals are scored.

### LN Analysis

In total, we identified 326 LNs for all treatments. In the interest of brevity, we have discussed here a selective but not exhaustive list of points. An unexpected finding from the LN analysis was the consistent regulation of Klf8 (Figure 7). Klf8 is a transcription factor that promotes cell cycle progression (Zhao et al., 2003) and can act as an oncogene by inducing proliferation and metastasis of cancer cells (Yan et al., 2015). In zebrafish, *klf8* is expressed in the CNS, where it is required for normal development of the cerebellum (Tsai et al., 2015). Interestingly, Klf8 was not identified as a DEG in any of the datasets (data not shown), suggesting that its activity is not regulated at the mRNA level. Indeed, Klf8 activity has been shown to be regulated by post-translational modifications (Wei et al., 2006; Urvalek et al., 2011). This suggests that Klf8 protein activity, but not mRNA abundance, may be a useful readout for neurotoxic or neuroactive substances. A zebrafish reporter line for Klf8 protein activity in the brain—for example, fluorescent protein expression driven by the Met promoter downstream of Klf8—might be suitable for this purpose (Gurevich et al., 2016). Interestingly, cerebellum neuronal development is abnormal in *rb1*-mutant mice and in rats exposed to Pb or Domo (Marino et al., 2003; Hogberg and Bal-Price, 2011; Mousa et al., 2015). This correlation suggests that Klf8 may mediate some of the above cerebellar phenotypes. As Klf8 is an oncogene, its activation by neurotoxins may increase the risk of cancer. Indeed, Pb exposure is associated with brain cancer (Anttila et al., 1996; Cocco et al., 1998; van Wijngaarden and Dosemeci, 2006), and preliminary data suggest that Domo can promote proliferation in cancer cells (Ayed et al., 2018). Furthermore, because antidepressants seem to inactivate Klf8, it is possible that antidepressants may help treat brain cancers. This does appear to be the case for some gliomas treated with antidepressants alone or in combination with other treatments (Liu et al., 2015; Shchors et al., 2015; Kushal et al., 2016), although a firm consensus has not been reached (Otto-Meyer et al., 2020). Further investigation into the role of Klf8 in the efficacy of antidepressants may help introduce a new class of treatment for brain cancers— an attractive option for drugs that are already on the market and can cross the blood brain barrier. Nodes downstream of Klf8 were consistently regulated together with Klf8. For example, in *rb1* mutants, activation of Klf8 led to activation of Met, which inhibited p53, increased cell proliferation, and decreased cell death. Because this sequence of events was reliably perturbed, it may make a good basis for a neurotoxicity adverse outcome pathway (AOP) (Vinken, 2013; Figure 7). Indeed, the network as a whole should make a useful resource for generating AOPs, because: it was constructed on the basis of evidence in literature; its edges have a direction; and it represents biological organization on multiple levels.

The response to Pb exposure indicated a switch from neurotoxicity at 72 h to general toxicity at 120 h. Autophagy and apical junction assembly were predicted to be strongly activated at 72 h and less so at 120 hpf, while cell death was activated at 120 hpf. Oxidative stress is known to disrupt the integrity of epithelial junctions (Rao, 2008). Thus, the present network analysis provides a possible scenario that the shorter Pb exposure triggered a protective response in the CNS, while the longer exposure led to oxidative stress and apoptosis.

Domoic acid is a neurotoxin that causes, among other symptoms, seizures in mammals and zebrafish (Tiedeken et al., 2005; Pulido, 2008). Domo mimics the neurotransmitter glutamate and can cause excitotoxicity by overstimulating the nerves (Chandrasekaran et al., 2004). Glutamate has been shown to downregulate Tsc2 expression in mouse cortical cultures via activation of mTor (mechanistic target of rapamycin) (Ru et al., 2012), and Tsc2 inactivation is associated with epileptic seizures (Zeng et al., 2011). Our LN analysis identified inhibition of Tsc2 activity in the Domo -treated group. Thus, a plausible mechanism for Domo -induced seizures in zebrafish is inhibition of Tsc2. Domo treatment increased dopaminergic neuron differentiation and the activity of dopamine receptors D2a and D2b. Although Domo has been reported to significantly decrease the number of dopaminergic neurons in culture (Radad et al., 2018), curiously, *in vivo* administration to rat brain via dialysis causes dopamine release (Alfonso et al., 2003). Our results suggest that zebrafish larvae respond to Domo similarly to rats and that the eventual reduction in the number of dopaminergic neurons is preceded by aberrant dopamine release.

### Limitations and Future Directions

We have shown the NPA value to be useful in comparing the magnitude of effect between chemicals, doses, and exposure durations. However, the NPA should not be interpreted as a standalone value for indicating neurotoxicity. The datasets tested here varied greatly in terms of the NPA, and most reached the threshold for statistical significance. Yet, most of the exposures in the transcriptomics studies used here were not reported to cause a neurotoxic phenotype. Therefore, the relationship between the NPA value and observable neurotoxicity (and consequently biological significance) is not apparent from current data. Furthermore, general toxicity does seem to perturb the network as is the case for the longer Pb exposure. Although the “o” statistic returned a not significant result for this treatment, we cannot exclude the possibility that general toxicity will result in significant NPA and perhaps will have a proportional response at a higher toxic and/or transcriptional response. In such cases the presence of general toxicity phenotypes in the sampled larvae should be considered when interpreting the scoring results.

Publicly available zebrafish transcriptomic data are not standardized. Variabilities in experimental design include chemical concentration, solvent use, exposure duration, age of the fish, light and temperature settings, presence or absence of the chorion, reported endpoints, and transcriptomics platforms. While this flexibility is great for basic research when answering a specific biological question, it is less suitable for toxicological and regulatory applications, as comparison of effects among different datasets is difficult. As an example, datasets that met our selection criteria for the current study (larval stage, neuroactive or neurotoxic chemical treatment, and three biological replicates per condition) display a considerable degree of heterogeneity. Dataset GSE129812 (suvorexant, imipramine, and 8beta treatments) was generated using larval brains and dataset GSE115720 (rb1 mutants) was generated using zebrafish larval heads, while all other datasets were generated using whole zebrafish larvae. Additionally, mRNA was collected between 3 and 8 dpf depending on the dataset. Although network scoring returns a significant NPA value for these treatments, it makes little sense to compare the transcriptome of 8 dpf brain to 3 dpf larva. Also, it is not yet clear whether comparison between networks is possible, since the data used to create each network, both the functional layer and the downstream transcripts, come from many different sources. Thus, at the moment, we encourage the use of NPA for comparison of treatments within a single dataset and in one network. On the other hand, the heterogeneity of the input data and biologically plausible, and in some cases consistent output results (e.g., klf8, rb1, p53), indicate that LN analysis can be a useful tool independent of source data. This raises the question of whether the network can be used as a filter to detect organ-specific changes in the transcriptome of the whole organism, thus bypassing manual dissection or FACS. One direct way to test this would be to sequence and score the transcriptomes of identically treated whole larvae and dissected brains.

The above points highlight an unmet need for systematic acquisition of transcriptomic data for toxicological assessment. To begin addressing this need, we are currently performing a larger chemical screening, where we are exposing larvae to chemical concentrations related to adverse outcomes (effect concentrations) under identical conditions, recording a standard set of phenotypes, and sequencing the mRNA on the same platform. Once collected and scored, this dataset should help connect gene expression data, NPA values, and observable phenotypes. Systematic acquisition of data should also be useful for network building. The current network was largely constructed using reports on single gene or protein expression. Although useful, this approach is low throughput. Transcriptomic data from zebrafish under or overexpressing specific genes at a specific stage would be a great resource to curate networks, fill in the current knowledge gaps, and populate the transcript layer.

The narrow scope of our network is both its strength and limitation. On the one hand, it allows assessment of chemical toxicity specifically in the organ of interest. On the other hand, it does not allow assessment of events that are not curated into the network. For example, we did not include essential processes for any developing organ, such as DNA replication or mitochondrial respiration, in order to avoid detection of non-CNS effects. Nor could we include events not documented in the literature. Consequently, the network covers only a subset of possible neurotoxic events. Additionally, the network is static as it represents only a narrow window during the development of the larva. One possible way to expand the scope of inquiry would be to create and score separate networks focused on different organs, biological processes, pathologies, and developmental stages. It is tempting to imagine that, in the near future, whole organisms will be curated into validated “network of networks” ready for analysis with a single transcriptomic dataset. Lastly, literature-based networks tend to be biased toward well-studied molecular players. To mitigate this, future networks could be populated with more diverse nodes by scoring relevant datasets against the entire iNode knowledgebase and connecting any new inferred nodes to the existing network model. The new hypotheses generated this way could be then verified experimentally, if literature to support these relationships doesn’t exist.

## Conclusion

We have developed a network-based method to test DNT in zebrafish larvae. This network recapitulates the biology of zebrafish and allows comprehensive toxicological assessment. The NPA represents high-dimensional transcriptomic data condensed into a single value, which helps compare chemicals and dose responses between similarly treated samples. The network identified Klf8 activity as a potential marker of neurotoxicity *in silico*. We suggest a prospective neurotoxicity AOP for further verification. Lastly, we propose plausible molecular mechanisms of DNT underlying the exposure to Pb and Domo. This approach should be useful to study developmental biology, drug discovery, and chemical toxicity.

## Data Availability Statement

The datasets presented in this study can be found in online repositories. The names of the repository/repositories and accession number(s) can be found below: http://causalbionet.com/Home/NetworkVisualization/#/!/networkGraph?ID=5ff47ba901dd761144521b5e, n/a.

## Author Contributions

AZ, CB, MT, JH, FM, and MP conceived the study. RL and MT constructed the network. AZ, MT, FM, and SG scored the network. RL, AZ, MT, and CB analyzed the results. All authors participated in manuscript preparation and review.

## Conflict of Interest

RL was an employee of Eawag at the time the experiments were performed, CB was an employee of Eawag, MT, JH, FM, SG, and MP are employees of Philip Morris International, and AZ is an employee of National Institute of Biology, Slovenia. The handling editor declared a past co-authorship with one of the authors JH.

## Footnotes

1 https://bel.bio

2 https://github.com/OpenBEL/language

3 https://github.com/OpenBEL/openbel-framework

4 https://pubmed.ncbi.nlm.nih.gov

5 https://www.ncbi.nlm.nih.gov/geo/

## Abbreviations

8beta: 8 β -(4 ′ -hydroxytigloyloxy)costunolide
DEG: differentially expressed gene
DNT: developmental neurotoxicity
Domo: domoic acid
Flu: fluoxetine
Imip: imipramine
LN: leading node
NPA: network perturbation amplitude
Ser: sertraline
Suvo: suvorexant.
