# Supplementary material for: Systems Toxicology Approach for Assessing Developmental Neurotoxicity in Larval Zebrafish
Source: Front Genet. 2021 Jun 15;12:652632. doi: 10.3389/fgene.2021.652632 (PMC8239408; doi:10.3389/fgene.2021.652632)
Supplement: Supplementary file 1 [file Data_Sheet_1.PDF]

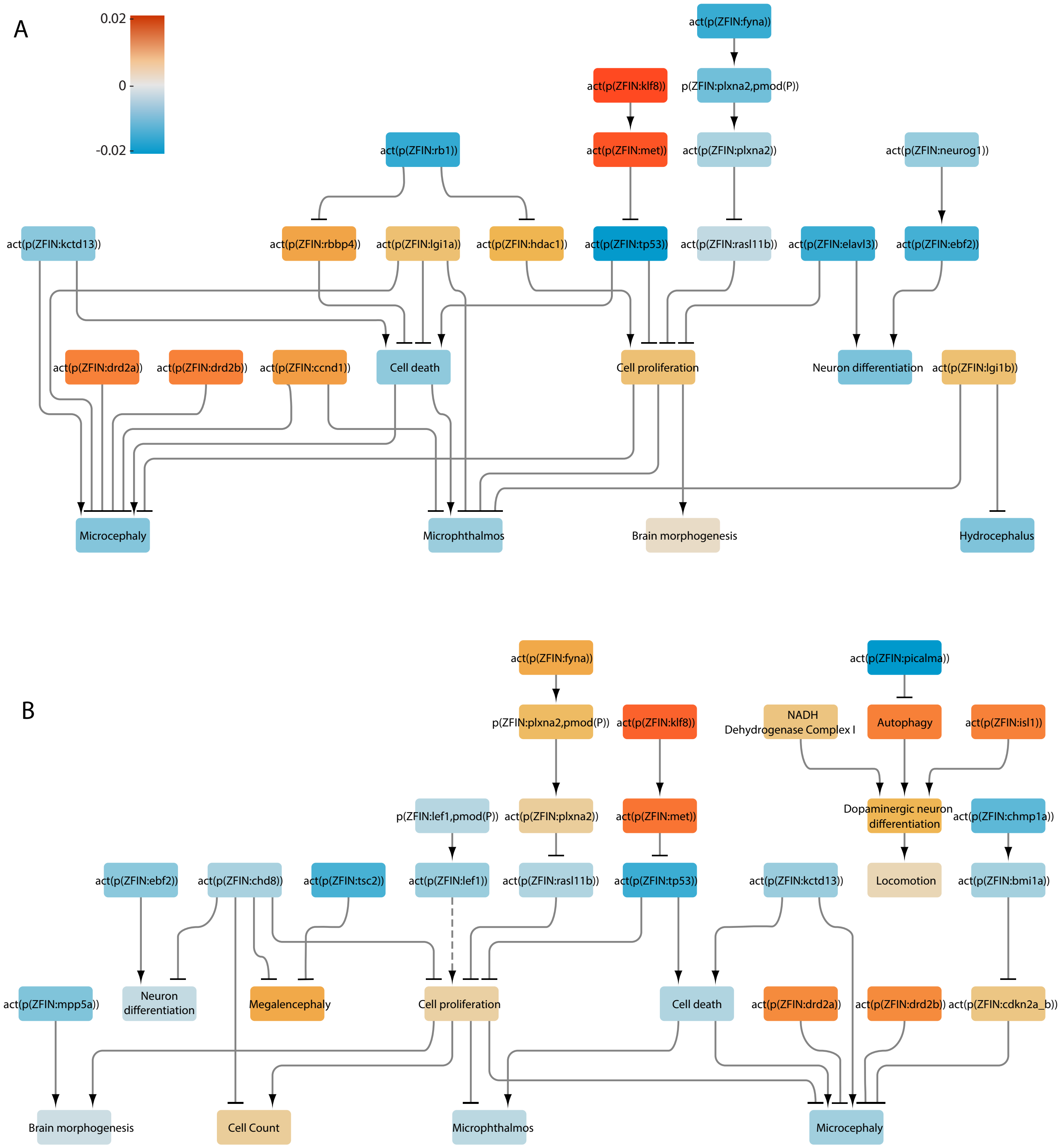

Figure S1. The neurotoxicity network scored with the transcriptomes of larvae treated with domoic acid. A. 3 dpf exposure. B. 7 dpf exposure. Increased and reduced activity for each node is color-coded according to the heatmap. Dotted lines represent indirect connections in the main network.

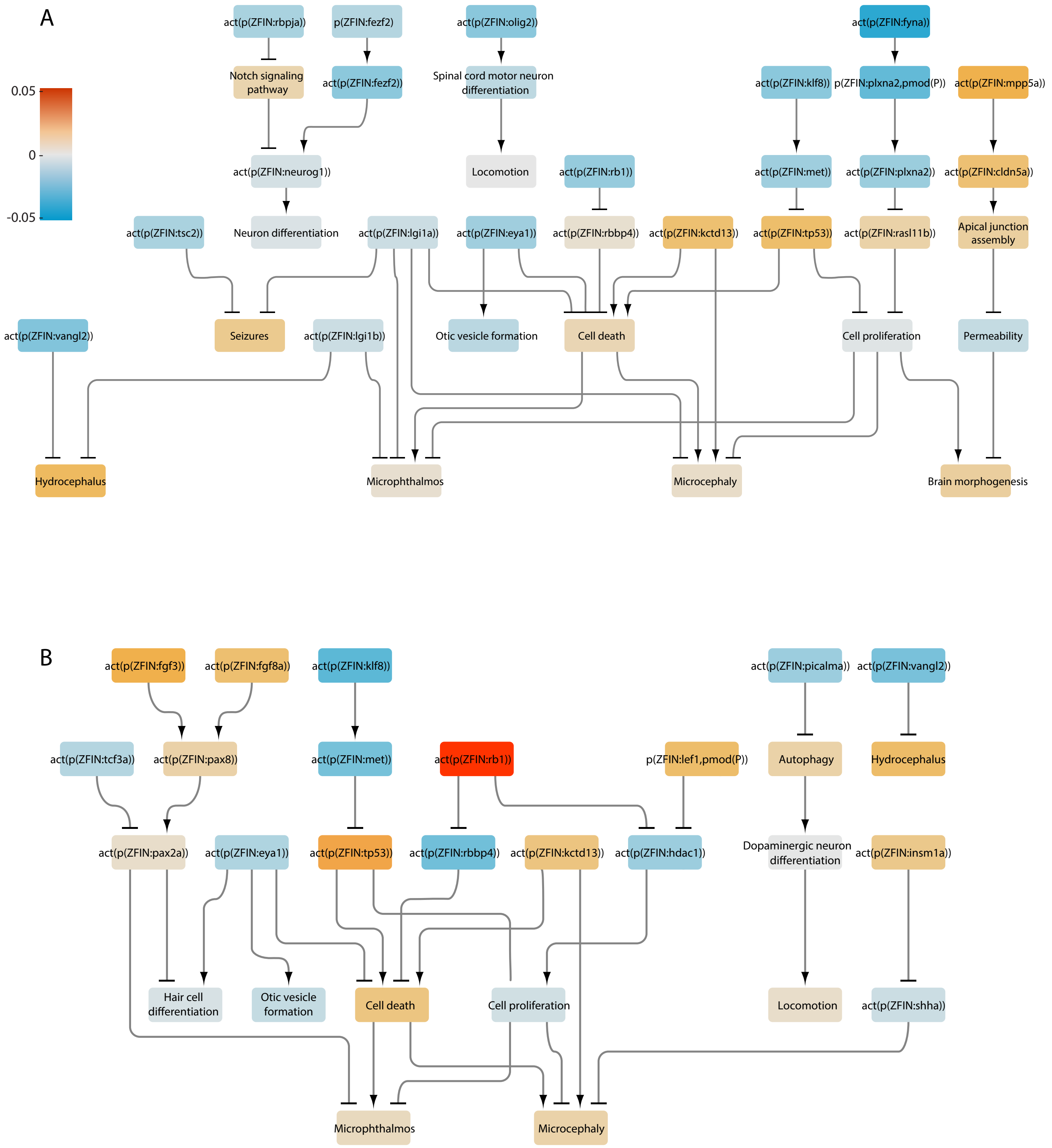

Figure S2. The neurotoxicity network scored with the transcriptomes of larvae treated with suvorexant (A) and imipramine (B). Increased and reduced activity for each node is color-coded according to the heatmap. Dotted lines represent indirect connections in the main network.

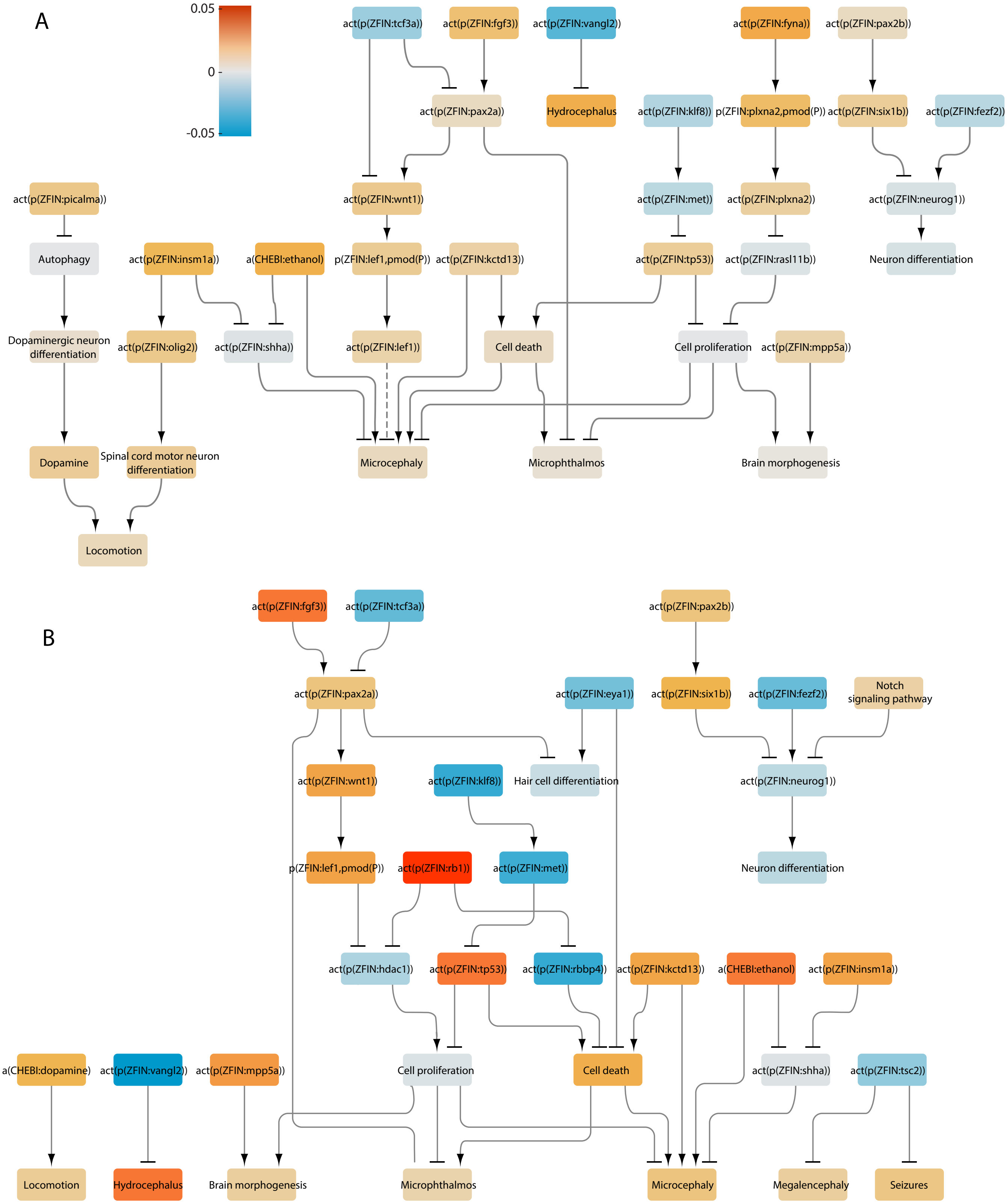

Figure S3. The neurotoxicity network scored with the transcriptomes of larvae treated with 8beta. A. 10 uM exposure B. 100 uM exposure. Increased and reduced activity for each node is color-coded according to the heatmap. Dotted lines represent indirect connections in the main network.
